# BLASSO: integration of biological knowledge into a regularized linear model

**DOI:** 10.1186/s12918-018-0612-8

**Published:** 2018-11-20

**Authors:** Daniel Urda, Francisco Aragón, Rocío Bautista, Leonardo Franco, Francisco J. Veredas, Manuel Gonzalo Claros, José Manuel Jerez

**Affiliations:** 1 Universidad de Cádiz, Departamento de Ingeniería Informática, Avda. de la Universidad de Cádiz n°10, Puerto Real, Cádiz, 11519 Spain; 2 Instituto de Investigación Biomédica de Málaga (IBIMA), Inteligencia Computacional en Biomedicina, Avda. Jorge Luis Borges n°15 Bl.3 Pl.3, Málaga, 29010 Spain; 3 Universidad de Málaga, Departamento de Lenguajes y Ciencias de la Computación, Bulevar Louis Pasteur, 35. Campus de Teatinos, Málaga, 29071 Spain; 4 Universidad de Málaga, Departamento de Biología Molecular y Bioquímica, Facultad de Ciencias, Campus Universitario de Teatinos, Málaga, 29071 Spain; 5 Universidad de Málaga, Plataforma Andaluza de Bioinformática, Parque Tecnológico de Andalucía, Calle Severo Ochoa 34, Málaga, 29590 Spain

**Keywords:** RNA-Seq, Biomarkers selection, Biological knowledge, Machine learning, Precision medicine

## Abstract

**Background:**

In RNA-Seq gene expression analysis, a genetic signature or biomarker is defined as a subset of genes that is probably involved in a given complex human trait and usually provide predictive capabilities for that trait. The discovery of new genetic signatures is challenging, as it entails the analysis of complex-nature information encoded at gene level. Moreover, biomarkers selection becomes unstable, since high correlation among the thousands of genes included in each sample usually exists, thus obtaining very low overlapping rates between the genetic signatures proposed by different authors. In this sense, this paper proposes BLASSO, a simple and highly interpretable linear model with *l*_1_-regularization that incorporates prior biological knowledge to the prediction of breast cancer outcomes. Two different approaches to integrate biological knowledge in BLASSO, *Gene-specific* and *Gene-disease*, are proposed to test their predictive performance and biomarker stability on a public RNA-Seq gene expression dataset for breast cancer. The relevance of the genetic signature for the model is inspected by a functional analysis.

**Results:**

BLASSO has been compared with a baseline LASSO model. Using 10-fold cross-validation with 100 repetitions for models’ assessment, average AUC values of 0.7 and 0.69 were obtained for the *Gene-specific* and the *Gene-disease* approaches, respectively. These efficacy rates outperform the average AUC of 0.65 obtained with the LASSO. With respect to the stability of the genetic signatures found, BLASSO outperformed the baseline model in terms of the robustness index (RI). The *Gene-specific* approach gave RI of 0.15±0.03, compared to RI of 0.09±0.03 given by LASSO, thus being 66% times more robust. The functional analysis performed to the genetic signature obtained with the *Gene-disease* approach showed a significant presence of genes related with cancer, as well as one gene (IFNK) and one pseudogene (PCNAP1) which a priori had not been described to be related with cancer.

**Conclusions:**

BLASSO has been shown as a good choice both in terms of predictive efficacy and biomarker stability, when compared to other similar approaches. Further functional analyses of the genetic signatures obtained with BLASSO has not only revealed genes with important roles in cancer, but also genes that should play an unknown or collateral role in the studied disease.

## Background

Personalized medicine in cancer aims to adapt diagnosis and treatment to patients on the basis of their environment and genes [1]. Hereof, large investments are being made in -*omics* technologies to sequence faster, cheaper and better [2, 3], what means more data to be used in clinical daily practice. Processing this huge amount of data is not, however, a trivial task, as they usually consist of a small number of samples (*n*) facing to the thousand of variables (*p*) that describe each sample (commonly known as large-*p*-small-*n* problems [4]). Concretely, in precision medicine, the search of genetic signatures still remains as a challenging task, and machine learning (ML) models and techniques have been recently used to develop predictive models in different areas [5–8], providing high performance rates in these large-*p*-small-*n* problems [9, 10].

Feature selection (FS) is one of the key procedures in the development of predictive models for complex human traits based on genomic data. In the literature, the available set of feature selection methods is grouped in three main categories: filter, wrapper and embedded procedures [11]. Independently of the FS procedure used, the goal is to identify a genetic signature with high prediction capabilities in a totally new and unseen test dataset, different to the one used to build the predictive model. Nevertheless, biomarkers selection becomes unstable as soon as the number of features gets larger (like in the *p*>>*n* scenario), specially due to the existing high correlation among the thousands of genes describing each sample [12]. In fact, Van’t Veer and colleagues [13] came up with a genetic signature of 70 genes that allows to predict clinical outcome of breast cancer with a good performance rate, and this signature is actually implemented in a commercial product known as the *MammaPrint test*. Two years later, Wang and colleagues [14] published a genetic signature of 76 genes that performed as well as the one discovered in [13], although only 3 genes were overlapped across both gene signatures. Finally, Venet et al. [15] showed that one can randomly pick any subset of genes that will significantly be associated with breast cancer outcomes. These results clearly indicate that it is necessary to impose some constrains to the ML models and FS techniques to overcome the huge variability observed.

Model interpretability is a second desired feature of ML models developed in biomedical contexts. Not only are we interested in developing ML models with high prediction capabilities, but also in being able to interpret the models themselves. Models’ interpretation have also been an active research topic in this area in the last years [16–18]. The interpretation of ML models allows researchers to perform biological and functional analysis based on the genetic signatures found to either confirm already existing knowledge of the studied disease or potentially discover new associations that may be worth to investigate further. Moreover, interpretable models have the advantage of identifying important genes that are predictive of the given outcome as well as identifying protective ones, thus possibly allowing to proceed with other relevant goals in personalized medicine, e.g. drug development to target specific genes of interest within a treatment, providing the right drug to the right patient [19, 20].

In this paper, the authors propose the BLASSO (Biological LASSO) predictive model, a new linear *l*_1_-regularization model that incorporates prior biological knowledge, from the PubTator public repository, to enrich the genes expression profiles in the human species. The proposal aims to quantify the importance of a given gene in the estimation of the predictive model based on the number of citations found in PubTator [21–23] for that particular gene. It is therefore expected that genes with a higher number of citations in PubTator will be more likely to be selected by the FS procedure and therefore included in the final genetic signature. Furthermore, a hypothetical less important gene will also be part of the genetic signature if this gene adds predictive value. Two different approaches for quantifying the importance of each gene are proposed in this paper (*Gene-specific*, *Gene-disease*) and their predictive performance and biomarker stability have been tested on a public RNA-Seq gene expression dataset for breast cancer (BRCA). Additionally, we show the advantages of our methodology in a controlled artificial dataset. Furthermore, the authors perform a functional analysis of the genetic signature found by the *Gene-disease* approach to discuss possible biological findings in the BRCA dataset.

The rest of the paper is organized as follows: the *Methods* section describes the datasets used within the experiments, the tools used to perform the functional analysis and the proposed methodology. The *Validation Strategy* section gives details of the performance measures and the validation strategy used to assess models’ performance. Next, the results obtained both in the artificial and BRCA datasets are shown in the *Results* section, followed by a *Discussion* section that provides a functional analysis and some discussions with respect to the genetic signatures found. Finally, the *Conclusions* section presents some conclusions obtained from this work.

## Methods

### Datasets

Two datasets were used to test the added benefits of the model proposed in this paper. Both datasets are high dimensional datasets and overall details are included in Table 1.

**Table 1:** Overall description of the datasets: number of samples (*n*), number of genes (*p*) and class distribution (*control*=0, *cases*=1)

Name	*n*	*p*	Controls	Cases
Artificial	1212	20021	583	629
BRCA	1212	20021	1013	199

On the one hand, a public RNA-Seq gene expression dataset of BRCA, freely available at The Cancer Genome Atlas (TCGA) website (https://cancergenome.nih.gov/) was used within the analysis. This dataset has already been batch-corrected and RSEM normalized [24]. In addition, we first removed those genes that do not show any expression across the samples (they do not add predictive value) and we performed a *log*_2_ transformation of the genes expression level to ensure they closely approximate to a normal distribution. After applying these pre-processing procedures, the final BRCA dataset consisted of *n*=1212 samples and *p*=20021 genes expression profiles describing each sample. Out of the 1212 samples, 1013 corresponds to controls (or alive patients) and 199 to cases (or patients who died from the disease). Therefore, the event of interest will be the vital status of a given patient (“ 0=*alive*", 1= “ *dead*") at a fixed time *t*.

On the other hand, another dataset with a synthetic outcome was created based on the real BRCA dataset. The idea behind this procedure tries to clearly know a priori the ground truth, i.e. which subset of genes are predictive of the outcome. This will provide us a controlled experimental design framework to test and confirm the advantages of using our proposal in this paper. In this sense, a subset of *k*=100 random genes out of the total number of *p*=20021 genes were first selected. Ideally, these genes should be the ground truth of the artificial dataset, thus representing the useful genes to predict the final outcome. Therefore, a synthetic outcome was then created by applying the sigmoid function described in the following equation:

1 $$  F_{{sig}}(\boldsymbol{x},\boldsymbol{\beta}) = \left\{\begin{array}{ll} 1, & \text{if} \frac{1}{1+e^{\boldsymbol{-x\beta}}}\geq 0.5\\ 0, & \text{otherwise} \end{array}\right.  $$

where the *k*=100 genes expression profiles and 100 randomly generated coefficients (*β*) sampled from a uniform distribution between [0,1] are given as input to the sigmoid function. Additionally, the class label of some samples were flipped to introduce some noise in the synthetic outcome created. In this artificial dataset, the ground truth is a priori known and the best solution that any linear model could get would be the identification of those *k* genes among the initial *p*, thus achieving the highest performance in terms of predictive accuracy.

### Functional analysis tools

Functional analyses for the discovered genes signatures within the BRCA dataset were performed using EnRichR (http://amp.pharm.mssm.edu/Enrichr/), WebGestalt 2017 (WEB-based GEne SeT AnaLysis Toolkit, http://www.webgestalt.org/), and the Ingenuity®; Pathway Analysis v 5.0 (IPA®;, QIAGEN, https://www.qiagenbioinformatics.com/) and the IPA client for Mac OSX. All analyses were performed using the gene symbols (HUGO gene names) as identifier and, when required, the beta coefficient as weighting value or an equivalent to fold change.

### Methodology

**Fig. 1:**
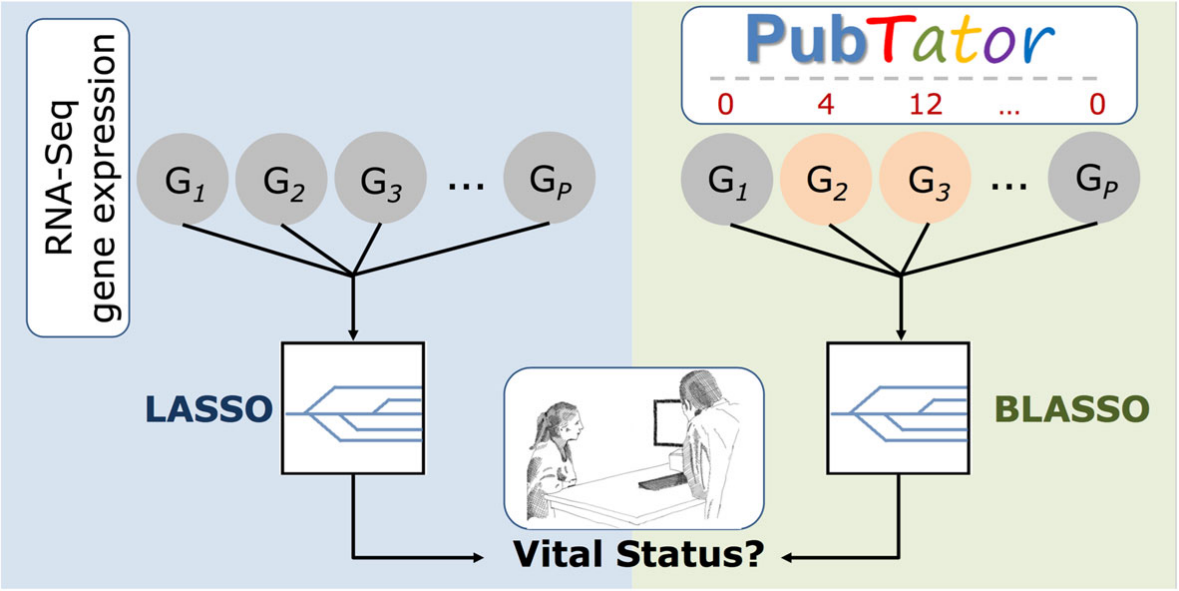
Methodology approach. Classic approach shown on the left side where a linear model with *l*_1_-penalty and homogeneous priors is used to predict the vital status of a patient given the RNA-Seq genes expression profile. On the right side, our methodology approach is described using prior biological knowledge obtained from public online resources to consider heterogeneous priors on the estimation of the *l*_1_-regularization model

This paper aims to include biological knowledge of the data domain into ML models, thus imposing constrains into the optimization search procedure. In the large-*p*-small-*n* scenario, linear models with *l*_1_-penalty term have been widely used as the simplest possible model with good prediction capabilities. Therefore, this work will try to somehow integrate biological knowledge into a *l*_1_-regularization model expecting it to outperform the classical approach. Figure 1 provides a high-level description of our methodology approach in comparison to the standard estimation of *l*_1_-regularization models.

#### The standard LASSO

LASSO is a widely known model [25] that adds an *l*_1_-penalty constrain to the objective function of a simple linear model. Let us assume a dataset represented as *D*={***x***_***i***_,*y*_*i*_}, with *i*∈{1..*n*} samples, ***x***_***i***_ representing the vector of *p* genes describing the *i*-th sample, and *y*_*i*_ being the class label. Then, Eq. 2 shows the objective function that is minimized under the LASSO approach and for a binary classification problem:

2 $$ J_{1}=\min_{\boldsymbol{\beta}} \enspace {\sum_{i=1}^{n}{(y_{i} - F_{{sig}}(\boldsymbol{x}_{\boldsymbol{i}}, \boldsymbol{\beta}))^{2}} + \lambda \sum_{j=1}^{p}{|\beta_{j}|}}  $$

where the function *F*_*sig*_ corresponds to the sigmoid function defined in Eq. 1.

This model tries to push as many coefficients (*β*_*j*_) as possible to zero unless a certain gene expression profile *x*_*j*_ is predictive of the vital status of a patient. LASSO models have been previously shown to work well in the large-*p*-small-*n* scenario being able to overcome overfitting issues. The amount of regularization applied is controlled by the hyper-parameter *λ* which takes values in the (0,1) range. When *λ* takes bigger values, then the *l*_1_-penalty term in Eq. 2 has a higher incidence in the whole objective function and, therefore, less genes will be retained by the model. The *λ* hyper-parameter is learned from data through cross-validation.

#### BLASSO: Biological LASSO

Despite the good performance achieved by LASSO in general problems, its main disadvantage when analyzing RNA-Seq data is that it considers homogeneous priors over the independent variables *x*_*ij*_, where *j*∈{1..*p*} genes, i.e. every single gene expression profile is equally treated and regularized in the optimization procedure. An extension of this model was later introduced in 2006 and named adaptive-LASSO [26], where heterogeneous priors were now considered. In this model, the *l*_1_-penalty term incorporates individual weights for each independent gene expression profile performing as well as if the true underlying model is given in advance. In this sense, Eq. 3 reflects the updated function that adaptive-LASSO tries to minimize for a binary classification problem:

3 $$ J_{2}=\min_{\boldsymbol{\beta}} \enspace {\sum_{i=1}^{n}{(y_{i} - F_{{sig}}(\boldsymbol{x}_{\boldsymbol{i}}, \boldsymbol{\beta}))^{2}} + \lambda \sum_{j=1}^{p}{\gamma_{j}|\beta_{j}|}}  $$

On the one hand, Eqs. 3 and 2 are equivalent when *γ*_*j*_=1, ∀*j*∈{1…*p*}. On the other hand, Eq. 3 would be identical to the objective function of logistic regression when *γ*_*j*_=0, i.e. no regularization is applied. This explanation shows that adaptive-LASSO could be understood as an intermediate model between logistic regression and a standard LASSO. Moreover, in the RNA-Seq context the ***γ*** vector could be used to measure the importance of each single gene expression profile. When a specific gene expression profile has its *γ*_*j*_ closer to zero, then the assigned coefficient *β*_*j*_ will have nearly no influence into the *l*_1_-penalty term, thus not being regularized by the model and, therefore, most likely this gene expression profile will be retained as part of the genetic signature discovered by the model. And vice versa, when a gene expression profile has its *γ*_*j*_ closer to one, then the assigned coefficient *β*_*j*_ will be an active part of the *l*_1_-penalty term, thus allowing the model to regularize and try to get rid of that gene expression profile without compromising the global error.

This work proposes to modify and enrich the adaptive-LASSO model by re-defining the ***γ*** vector in such a way that prior biological knowledge of the data domain can be integrated in the model. Given a particular gene expression profile *x*_*j*_, authors propose to re-define the individual penalty factor *γ*_*j*_ of the gene as shown in Eq. 4:

4 $$ \gamma_{j}=\left(\frac{1}{\#{cites}_{j}+1}\right)^{\epsilon}  $$

thus resulting in the objective function shown in Eq. 5 that BLASSO will try to minimize:

5 $$  \min_{\boldsymbol{\beta}} \enspace {\sum_{i=1}^{n}{(y_{i} - F_{{sig}}(\boldsymbol{x_{i}}, \boldsymbol{\beta}))^{2}} + \lambda \sum_{j=1}^{p}{\left(\frac{1}{\#{cites}_{j}+1}\right)^{\epsilon}|\beta_{j}|}}  $$

Assuming that it is possible to get the number of citations for a given gene expression profile, this definition will behave exactly as explained before. Let us consider a gene for which there are no citations available, i.e. an a priori non-relevant gene expression profile according to the literature. Then, by definition its corresponding *γ*_*j*_ value would be 1 and, therefore, BLASSO will try to regularize and get rid of that gene pushing its *β*_*j*_ coefficient to zero whenever this gene has no predictive value in the analyzed dataset. Instead, let us now consider a very relevant gene for which there are hundreds of citations in the literature. In this case, the corresponding *γ*_*j*_ value would be a very small number close to zero, thus the value of its *β*_*j*_ coefficient will have nearly no influence into the *l*_1_-penalty term and, most likely, this gene expression profile will be retained as part of the final genetic signature discovered by the model.

BLASSO has two hyper-parameters to be tuned, *λ* and *ε*. The first one, *λ*, is the regularization rate of the standard LASSO problem. The second one, *ε*, is a hyper-parameter that takes values within the range (0,1], which basically helps to control the smoothness of the individual-gene regularization applied by the model. The motivation of this hyper-parameter arises from situations where an specific gene may have thousands of citations in the literature, i.e. an a priori very relevant gene expression profile. As it has been highlighted before, by definition its *γ*_*j*_ value would be very close to zero and, most likely, the gene will be finally retained by BLASSO in the genetic signature. If many more gene expression profiles are under the same situation, then BLASSO would be getting closer to the over-fitting issue faced by logistic regression as there will be many genes that BLASSO will most likely not regularize and retain in the genetic signature. A deeper analysis of this hyper-parameter within the BRCA dataset context is provided in the *Results* section. Both hyper-parameters, *λ* and *ε*, are learned from data through cross-validation.

At this point, the question for which an answer is needed would be: are there any public online resources available where an estimation of the importance of each individual gene expression profile could be retrieved? One could think of developing a data mining procedure that extracts relevant information of interest from PubMed. However, there are some recent works that have previously addressed this issue. In 2016, Andrade-Navarro et al. [27] published and online tool that uses an automatically built dataset of more than 63 thousand gene-disease associations defined as statistically significant co-occurrences of genes and diseases in annotations of biomedical citations from PubMed. Wei et al. [21–23] proposed in 2013 a web-based tool named PubTator for accelerating manual literature curation through the use of advanced text-mining techniques. In particular, PubTator stores all the PubMed IDs of published articles, the set of genes referenced on each of the articles, and the disease or diseases involved in the corresponding study. In this work, PubTator was chosen as the online resource to be used to get prior biological information of the data domain as it is a widely cited tool of reference and is more mature than the one recently provided in [27], thus being an a priori more robust framework to test the benefits of the proposed model BLASSO.

Furthermore, this paper proposes two different approaches to construct the ***γ*** vector based on PubTator. Both approaches are based on counting gene citations occurrences, although they differ on the scope literature taken into account for this purpose. Next, a description of the two approaches considered in this work is provided:

- ***Gene-specific***: for a given gene symbol *x*_*j*_, this approach will count the number of articles where this gene has been cited, independently of the studied context, to generate the corresponding individual penalty *γ*_*j*_ using Eq. 4.
- ***Gene-disease***: for a given gene *x*_*j*_ and a given disease of interest, e.g. BRCA, this approach will count the number of articles in the BRCA context where this gene has been cited to generate the individual penalty *γ*_*j*_ using Eq. 4. This approach adds the benefits of not taking into account articles where the gene was cited in a different context, thus not boosting the importance of genes that a priori are not relevant in the BRCA context.

Both approaches are valid ways of including prior biological knowledge into BLASSO either using PubTator or any other online resource that allows to retrieve the number of citations found in the literature for a particular gene symbol. However, the second proposed approach *Gene-disease* would make more sense when performing a functional analysis since a priori only genes already known to be associated with the studied disease (BRCA) will have an individual penalty *γ*_*j*_ different to 1. Therefore, the final genetic signature should contain many more relevant genes in the BRCA context and possibly a few that have not been associated to BRCA yet but that they turn out to add predictive value in the analyzed dataset.

## Validation Strategy

**Fig. 2:**
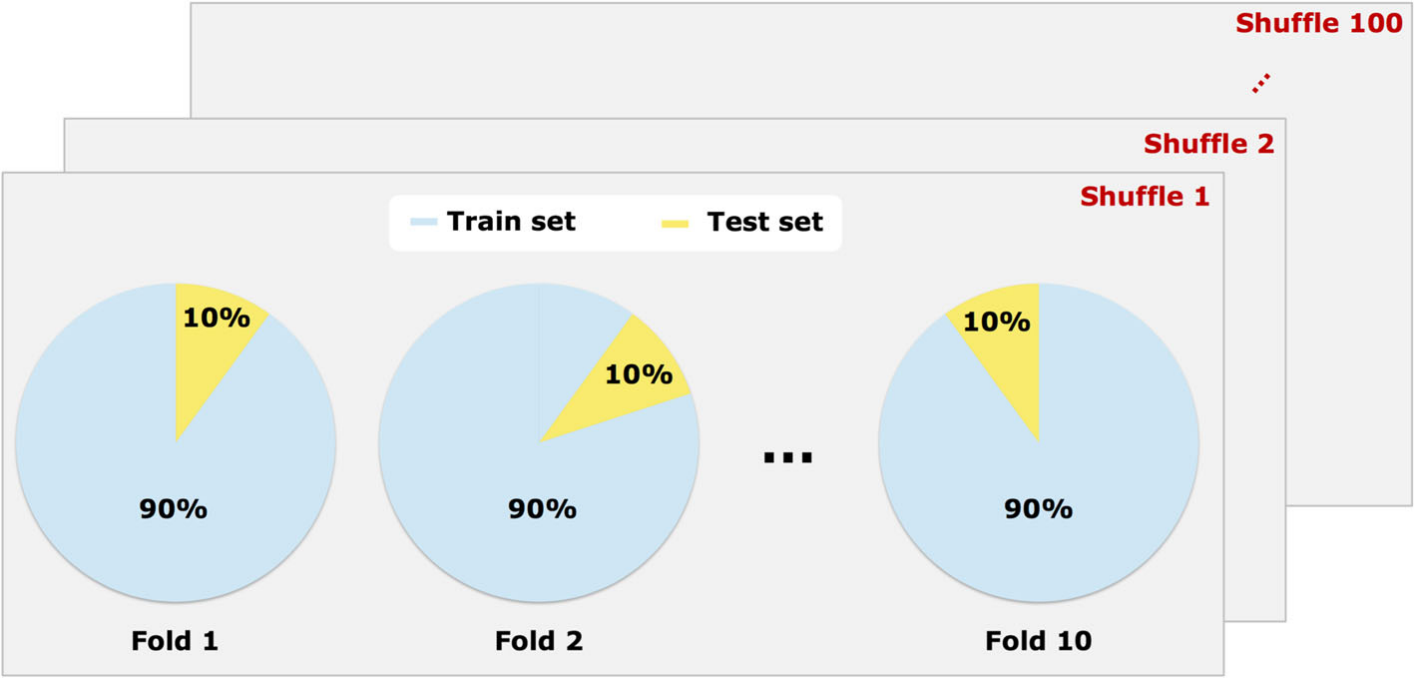
Validation strategy. 10-fold cross-validation scheme where train and test folds are iteratively rotated. A single iteration uses 90% of the data to fit the models (light blue) and 10% of the data to test their performance. The fold partitioning procedure is repeated 100 times to create different folds partitioning of the input data

In the analysis carried out in this paper, a well-known validation strategy was used to test the performance of the proposed model in new unseen data. Particularly, 100 repetitions of *K*-fold cross-validation (*K*=10) were executed as depicted in Fig. 2. *K*-fold cross-validation is a strategy that partitions the data into *K* non-overlapping folds of equal sizes. Models are fitted to data using samples contained in *K*−1 train folds and their performance is then tested in the outer test fold left out from the estimation process. This procedure is iteratively repeated rotating the train and test folds to finally provide an average model performance on test folds. Additionally, the strategy considered in this work repeats this procedure 100 times in such a way that the 10-fold partitioning of each repetition are different one from the other, thus ensuring that no bias is introduced in the analysis due to an specific fold partitioning randomly sampled. As both the *LASSO* and *BLASSO* models need some hyper-parameters to be learned, a second level of cross-validation is introduced within the *K*−1 train folds in order to pick the best hyper-parameter settings.

The Area Under the Curve (AUC) was the chosen measure to test the performance of the models as the BRCA in particular is highly imbalanced containing 199 cases and 1013 controls (see Table 1). Moreover, not only are we interested in analyzing the performance of the models but also in analyzing the stability of the genetic signatures obtained. In this sense, authors proposed to measure this stability calculating a robustness index (RI) defined as follows:

6 $$  RI=average\left(RI^{(1)}, \enspace... \enspace, RI^{(100)}\right)  $$

and the robust index for a single repetition is defined by the following equation:

7 $$  RI^{(rep)}=\frac{\#\left(intersection({genes}_{1}, \enspace... \enspace, {genes}_{10})\right)}{average(\#{genes}_{1}, \enspace... \enspace,\#{genes}_{10})}  $$

where the numerator corresponds to the number of overlapping genes across the 10 folds of the cross-validation in the specific repetition, and the denominator measures the average number of retained genes across the folds in the considered repetition. The higher and closer to 1 the RI is, the more robust the solution would be, as a larger overlap will be found in the genetic signatures.

## Results

The whole analysis was implemented under the R software using the package “glmnet” [28] which includes a nested cross-validation scheme in which the regularization rate *λ* is automatically adjusted. Additionally, extra functionality was developed within this package to automatically adjust the value of the hyper-parameter *ε* related to the smoothness of the individual gene regularization.

### Artificial data

The artificially generated data set (see details in the *Materials and Methods* section) was further used for testing several LASSO and BLASSO models under different conditions. The main objective of these experiments was to mainly get the feeling that the proposed model, BLASSO, works as it is expected. In this sense, just one repetition of 10-fold cross-validation was executed as it is enough to see the added benefit of BLASSO independently of the variance of the model. In Table 2 the results obtained are shown for each of the models that are described below:

- *LASSO*_200_: standard LASSO model with homogeneous priors fitted to the *k*=100 genes used to generate the synthetic outcome plus another 100 genes randomly selected.
- *LASSO*_2000_: similar to the previous one but now with the addition of 1900 randomly selected genes on top of the *k*=100 genes used to generate the synthetic outcome.
- *LASSO*_20021_: similar to the previous ones but now fitted to the entire dataset, thus using the whole 20021 set of genes.
- *LASSO*_19921_: standard LASSO model with homogeneous priors fitted to the entire dataset after removing the *k*=100 genes used to generate the synthetic outcome.
- *E*_1_- *BLASSO*_20021_: BLASSO model with penalty factors set to *γ*_*j*_=1 for the 19921 genes not used to generate the synthetic outcome, and *γ*_*j*_=*α*, where *α*∼*unif*(0,1), for the *k*=100 genes that were used to generate the synthetic outcome. The hyper-parameter *ε* was set to 1.
- *E*_2_- *BLASSO*_20021_: similar to the previous one but using *γ*_*j*_=0 for the *k*=100 genes used to generate the synthetic outcome.

**Table 2:** Average test data results obtained in a synthetic data set using different models. Values for the Area Under the Curve (AUC), average number of selected genes (#genes), and average number of genes overlapped with the *k*=100 genes used to generate the synthetic outcome (#genes*) are shown

Model	AUC	#genes	#genes*
*LASSO*_200_	0.9920±0.00	137.9	88.7
*LASSO*_2000_	0.9504±0.02	230.7	56.5
*LASSO*_20021_	0.9325±0.03	286.8	24.8
*LASSO*_19921_	0.8972±0.03	254.8	0
*E*_1_- *BLASSO*_20021_	0.9805±0.01	133.7	66.9
*E*_2_- *BLASSO*_20021_	0.9923±0.01	100	100

The results shown in Table 2 confirm what was initially expected from the application of the proposed models to the artificially generated data set. The first three settings are showing how the complexity of the analysis increases when more genes are added to the input dataset (the AUC drops from 0.9920 to 0.9325). Moreover, the average number of selected genes within the *k*=100 genes used to generate the synthetic outcome (column #genes*) reflects how unstable is the FS procedure when the aim is to identify the ground truth in wider datasets (larger number of input variables), as the value of overlapped genes drops from 88.7 to 24.8 genes. In addition, the fourth setting supports the statement made in [15], where a relatively good performance (AUC=0.8972) can be achieved even if the *k*=100 genes were not included in the input dataset for the analysis. In this sense, it shows that it is almost always possible to find a different genetic signature with high predictive accuracy when higher correlations exist among genes. Finally, the last two settings show the advantages of using the proposed model in this paper, BLASSO, which incorporates prior biological knowledge into the *l*_1_-penalty term. In concrete, the fifth setting simulates a possible scenario where the *k*=100 genes used to generate the synthetic outcome are less regularized (*γ*_*j*_=*α*, where *α*∼*unif*(0,1)), assuming that these genes are more relevant according to information from citations resources. In this setting, the AUC goes up to 0.9805 in contrast to the value of 0.9325 where homogeneous priors were used in the *l*_1_-penalty term. At the same time, the average number of overlapped genes with the *k*=100 genes used to generate the synthetic outcome is 66.9 instead of 24.8 found for the third setting. The last case consider (*E*_2_-BLASSSO_20021_) shows an ideal scenario, where *γ*_*j*_=0 for the *k*=100 genes used to generate the synthetic outcome according to citation resources (in practice, this may be unachievable). In this ideal case, it is possible to recover the original genetic signature (the ground truth, #genes* =100) and obtain the best performance (AUC=0.9923).

### BRCA data

**Fig. 3:**
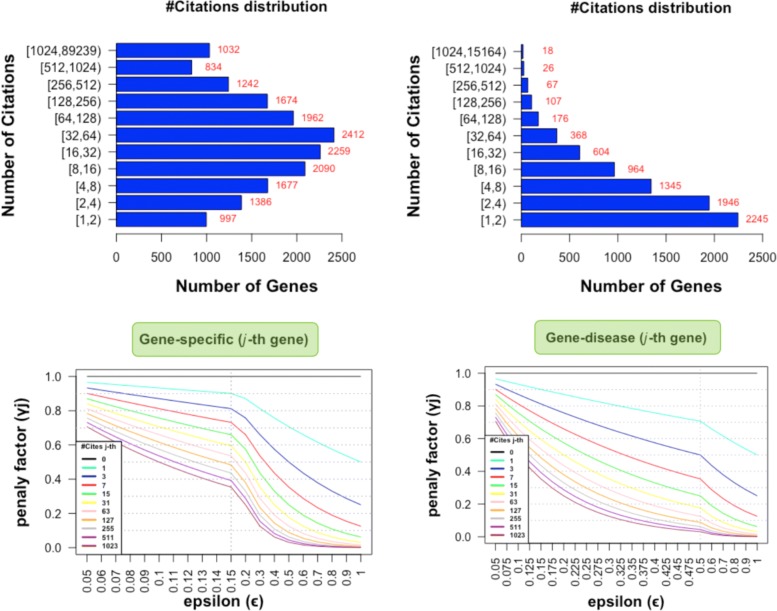
Citation distribution and role of the hyper-parameter *ε* in the penalty term. Graphics on the columns show on top, the distributions of citations for the whole set of genes, for the *Gene-specific* (left) and *Gene-disease* (right) approaches. The graphs at the bottom show the value of the penalty factor *γ*_*j*_ as a function of the value of *ε* (note the discontinuity of the scale in both graphs indicated by a vertical dotted line)

Before going into details of the performance results obtained in the experiments, a deeper analysis of the role of the hyper-parameter *ε* was performed using the BRCA data. In this sense, Fig. 3 shows some detailed graphs regarding the gene citations distribution under the two considered approaches (*Gene-specific* and *Gene-disease*) as well as the relationship between the penalty factor *γ*_*j*_ with respect to the value of *ε*. The top left figure shows the distribution of citations for the genes in the *Gene-specific* approach, observing that a large number of them have been cited several times in the literature. As these fact will lead these genes to be preferentially selected, smaller values of *ε* were tested for this approach as to reduce this effect. For the *Gene-disease* case, the situation is a bit different as there are fewer genes getting so many citations. As such, some larger values of *ε* were tested more in detail for this approach. The graphics at the bottom of Fig 3 shows the value of the penalty factor (*γ*_*j*_) as a function of the value of *ε*. The values of *ε* indicated on the *x*-axis in both graphs were the values that have been tested in the internal cross-validation simulations to learn the best *ε* value given the input BRCA data.

**Fig. 4:**
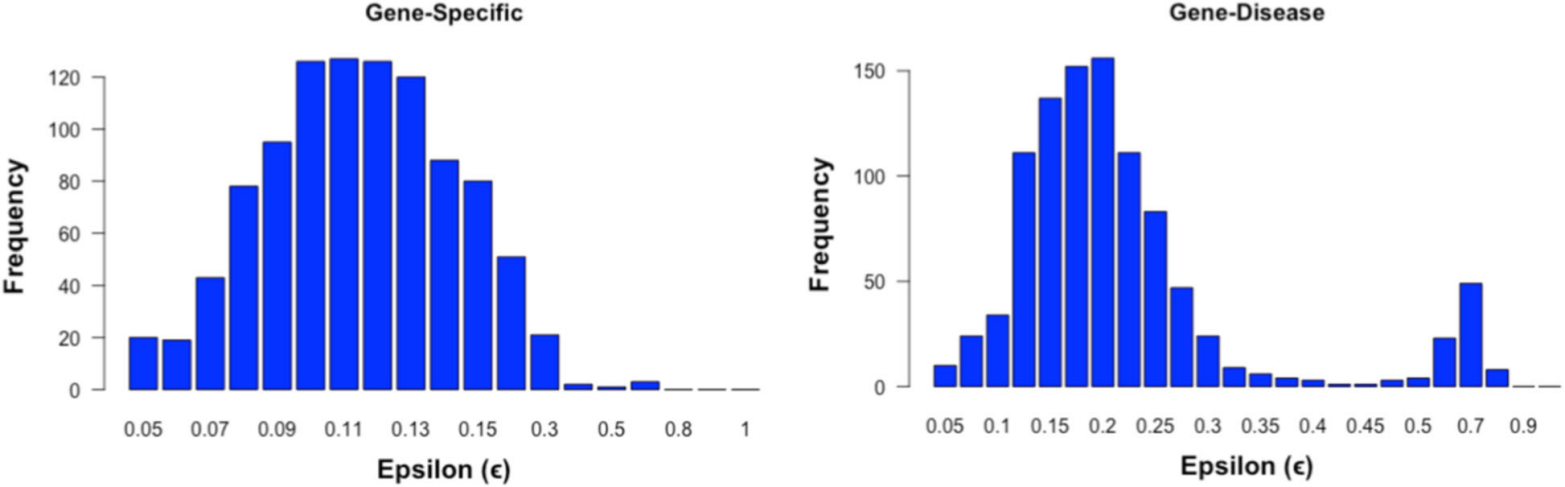
Hyper-parameter *ε* distribution. Frequency distribution of the *ε* values learned throughout the experiments for both cases considered: *Gene-specific* and *Gene-disease*

Furthermore, the hyper-parameter *ε* affects the amount of penalization that is included in BLASSO for a given gene expression profile, and is learned through nested cross-validation within the 100 repetitions of 10-fold cross-validation as described in the *Validation Strategy* section. Figure 4 shows the frequency distribution of different *ε* values learned for both cases considered: *Gene-specific* and *Gene-disease*. The values obtained for the *Gene-specific* case, following a bell-shaped distribution with peaks at *ε*=0.11, confirm the criterion chosen for the set of values tested, while for the *Gene-disease* approach the situation is slightly different, as a second peak is obtained around *ε*=0.7, thus indicating that a finer analysis could be done in a region around this value.

Regarding the predictive models used within the analysis, the BRCA RNA-Seq dataset was analyzed trying to obtain predictions of patients’ vital status, comparing results from three different settings: (i) standard LASSO with homogeneous priors as baseline model, (ii) BLASSO with heterogeneous priors obtained by the *Gene-specific* approach, and (iii) BLASSO with heterogeneous priors obtained by the *Gene-disease* approach. Each of these models were evaluated following the strategy described in the *Validation Strategy* section (100 repetitions of 10-fold cross-validation), and the results obtained for each of them are shown in Table 3. The first column of the table identifies each of the three setting tested. Then, the AUC values with the 95% confidence intervals (CI), the number of genes retained by the models (#genes), the robustness index (RI) as defined in the previous section, the computational time in minutes (time) and the significance value (*p*-value) provided by a Wilcoxon signed rank test [29–31] for the comparison of the alternative models with the baseline case in terms of the AUC are shown. With respect to the CI provided, Bengio et al. [32], showed in their work that CI should be taken carefully as they proved that there is no unbiased estimator of the variance of *K*-fold cross-validation, thus possibly representing over-optimistic results.

**Table 3:** Average test data results obtained in the BRCA RNA-Seq dataset for the baseline (standard LASSO with homogeneous priors) and BLASSO with the two proposed approaches. The Area Under the Curve (AUC), average number of selected genes (#genes), robustness index (RI), computation time (mins.) and significance *p*-value are shown

Model	AUC	#Genes	RI	Time	*p*-value
*Lasso*	0.65 [0.63, 0.68]	283.84±28.73	0.09±0.03	21.6	-
*Gene-specific*	0.7 [0.66, 0.71]	238.73±19.31	0.15±0.03	2341.83	<2.2×10^−16^
*Gene-disease*	0.69 [0.66, 0.71]	226.59±20.01	0.1±0.04	2784.32	<2.2×10^−16^

In terms of the AUC and for both approaches proposed in this paper (*Gene-specific*, *Gene-disease*), it is possible to find a parameterization of the models for which the baseline estimation is outperformed. In concrete, the *Gene-specific* approach obtained an average AUC value of 0.7 while the *Gene-disease* approach got an slightly lower AUC value of 0.69. Nevertheless, both values are higher than 0.65, the AUC value obtained with the standard LASSO model with homogeneous priors. It may not look an impressive improvement, but a difference of 0.04 is still quantitatively a good result taking into account that both LASSO and BLASSO are simple models that assume a linear relationship between the independent variables and the outcome, thus not capturing all possible non-linearities existing in the data. In addition, the improvement obtained was achieved using in average less genes than the ones retained by LASSO (283.84 genes compared to 238.73 with the *Gene-specific* approach and 226.59 with the *Gene-disease* one). Moreover, the two proposed approaches are highly statistically significant (*p*-value <2.2×10^−16^) according to a Wilcoxon signed rank test.

Regarding the stability of the genetic signatures found, both proposed approaches outperform the baseline model in terms of the robustness index defined. The *Gene-specific* approach obtains a RI of 0.15 compared to 0.09, thus being 66% times more robust. Further, the *Gene-disease* approach was found to be less robust, achieving a RI of 0.1 similar to the value found for the baseline LASSO model. It is worth noting that a robustness value of 0.15 indicates that on average 15 genes out of 100 are common on different executions of the algorithm, noting that for example in previous works [13] and [14], only 3 out of 70–76 genes were respectively overlapped among the genetic signatures provided (less than 5% overlap). If we measure the stability of the genetic signature across repetitions, the *Gene-specific* approach remains being more robust than the *Gene-disease* approach (0.013 of the first model compared to 0.004 of the second one). Despite the positive results found, one negative aspect of the introduced approaches regards the computational times needed, as they are approximately 100 times larger than the time required for the execution of the baseline LASSO model. However, standard existing software was used to carry out the analysis since optimizing the estimation procedure was not the scope of this paper. Under a first cross-validation level which leaves a test set apart (not used to estimate the LASSO or BLASSO models), the BLASSO model requires two additional levels of cross-validation: one is added in our implementation to learn the hyper-parameter *ε*, and another one added by the R package glmnet to learn the hyper-parameter *λ*. This logically increases the time to run the analysis using BLASSO but optimizing BLASSO was out of the scope in this paper. Therefore, further work could be done in this line to reduce the time required to estimate the BLASSO model.

## Discussion

**Fig. 5:**
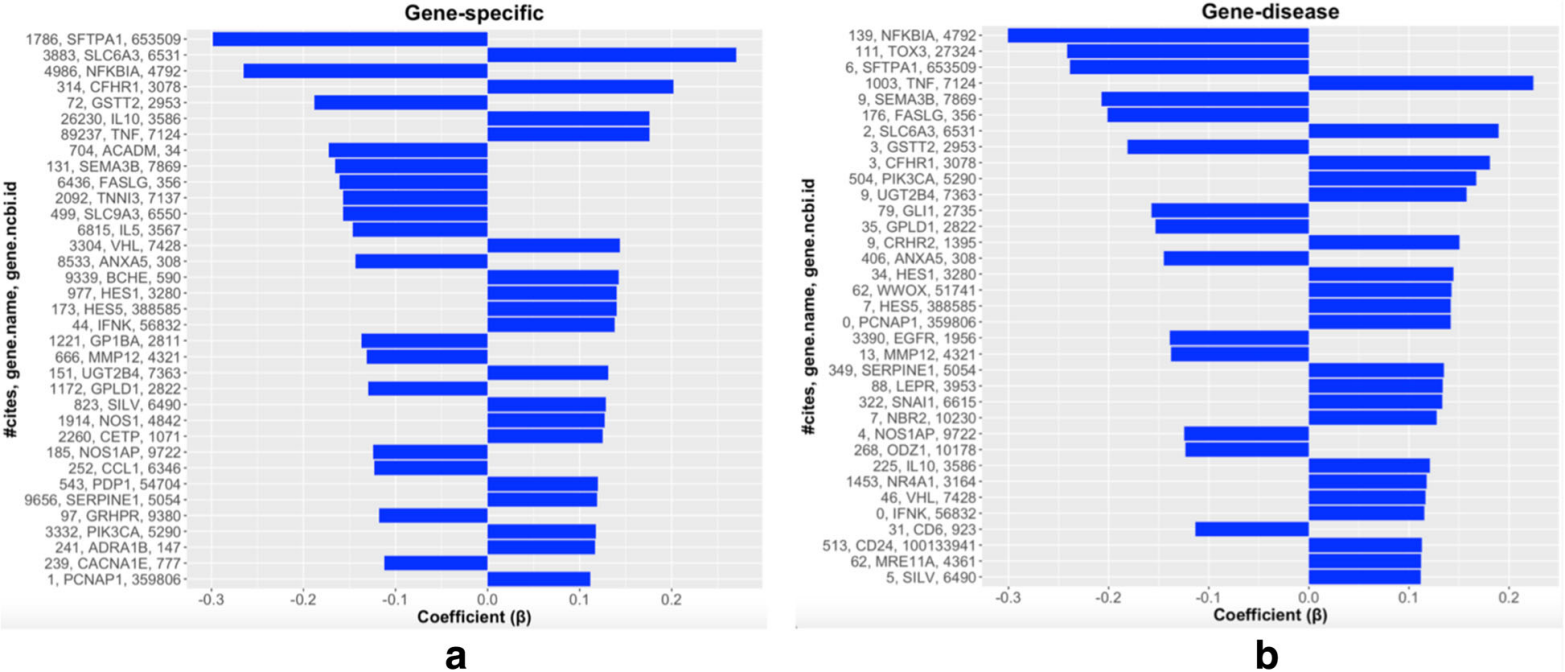
Global models’ summary. Summary of the top-35 selected genes according to the |*β*| coefficients of, (a) A genetic signature of 222 genes obtained using BLASSO with the *Gene-specific* approach; (b) A genetic signature of 219 genes obtained using BLASSO with the *Gene-disease* approach. Both models were estimated using the complete dataset

Parametric models and linear models in particular have the advantage of easier interpretation of the estimated model, thus opening the possibility of validating the gene signatures with external functional analysis tools. In this sense, both the *Gene-specific* and *Gene-disease* approaches were used to estimate BLASSO to the complete BRCA dataset. Figure 5 shows a sorted list of the top-35 genes that contribute more to predict the outcome in both genetic signatures. The higher a gene appears in the figures, the more it contributes to predict the vital status of a patient. In addition, those genes highly expressed with positive coefficients (bars positioned to the right) will increase the chances of not surviving while genes highly expressed with negative coefficients (bars positioned to the left) are protective of not surviving.

At this point, it is desirable to perform a functional analysis of these gene signatures to try to validate possible biological findings within the models. For this purpose, authors decided to focus on the 219 genes obtained as signature with the *Gene-disease* approach since it a priori takes into account genes known to be related with the studied disease. In first instance, EnRichR revealed that the main diseases based on OMIM were breast cancer, lung cancer, and colorectal cancer; based on KEGG, prostate and general cancer pathways; based on Reactome, signalling pathways; based on Panther, apoptosis, hypoxia, and P53 and VEGF pathways. When the beta coefficient is included to weight genes, the most results become apoptosis and pancreatic cancer, as well as other signalling pathways. An equivalent analysis using WebGestalt revealed liver carcinome (*p*-value=3.9×10^−9^), mammary neoplasm (*p*-value=3.9×10^−9^), followed by adenocarcinoma (*p*-value=2.1×10^−6^) and neoplasm metastasis (*p*-value=1.2×10^−4^). As expected, the main diseases involved in the gene signature were associated with cancer, and also apoptosis (cell death).

**Fig. 6:**
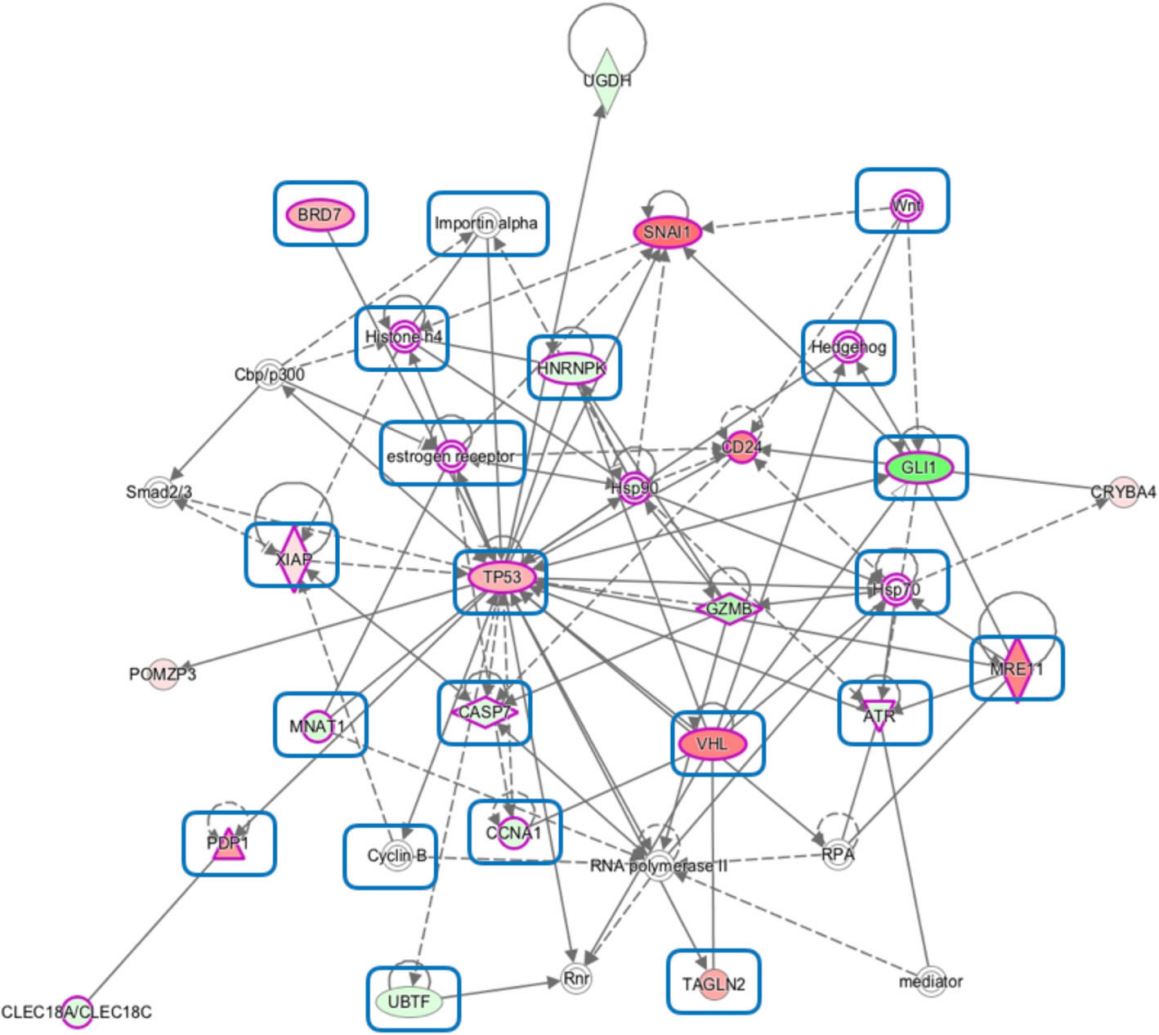
Cell Death and Survival, Cancer, Neurological Disease. Most significant functional network obtained with the 219 signature genes of the *Gene-disease* strategy. It corresponds to cell death and survival, cancer, and neurological disease biological functions. Node fill colour intensity correspond to the value level of beta (green for negative, red for positive); white nodes are those genes not appearing in the gene signature. Nodes delimited by a purple line correspond to cancer; those surrounded by a blue box correspond to cell death. Solid grey lines correspond to direct relations, while dashed lines correspond to indirect relations

Trying to obtain more details on the importance of each gene, the 219 genes were analyzed for their implication in the biological functions using IPA®; v5.0. A total of 19 different network were obtained, most of them corresponding, as expected again, to biological functions related to cancer, cell death, and signaling, but also with cellular development and cellular compromise. The most significant one is the first network, corresponding to cell death (including apoptosis) and survival, cancer, and neurological disease (Fig. 6). A total of 21 genes from the signature appeared in this network, most of them related both with cancer and cell death, which is consistent with the information obtained with EnRichR and WebGestalt (see above). This supports the idea that this signature is comprised of genes involved, directly or collaterally, in the analyzed disease. The key-role genes of this network are TP53, that is at the 66^*th*^ position of the signature, together with GLI1 (12^*th*^), and SNAI1 (24^*th*^), in collaboration with other prominent genes, such as, VHL (30^*th*^), CD24 (33^*th*^), MRE11 (34^*th*^).

**Fig. 7:**
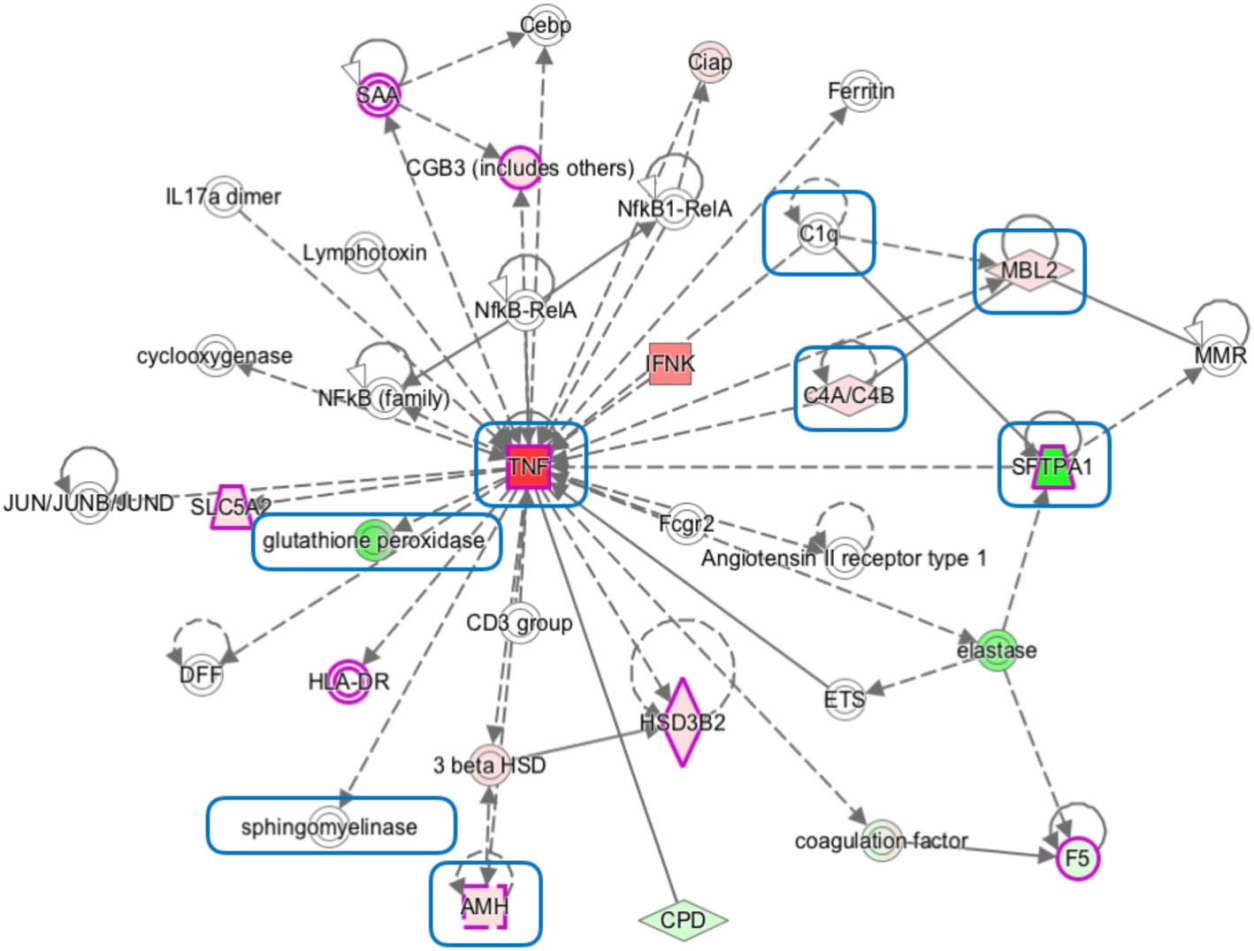
Cell Death and Survival, Infectious Disease, Cellular Compromise. Functional network obtained with the 219 signature genes of the *Gene-disease* strategy corresponding to cell death and survival, infectious diseases, and cellular compromise. Colours are as in Fig. 6

However, there are some genes, such as PCNAP1 (pseudogene 1 of the proliferating cell nuclear antigen in human [33]) and IFNK (a cytokine that imparts cellular protection against viral infection in a species-specific manner) without literature relation to cancer that appear in a relevant position regarding their beta value (19^*th*^ and 31^*th*^ position, respectively). The only IPA network containing IFNK corresponds to cell death and survival, infectious diseases, and cellular compromise (Fig. 7), where 11 genes forming the network were present in the signature. Cancer and cell death are highlighted in Fig. 7 to reveal that most genes are involved only in one of these functions (in contrast to network of Fig. 6, where most genes are involved in both functions). Interestingly, this network is the only one with two top-five genes. More in detail, it contains only one key node, TNF-alpha, which is the 4^*th*^ gene of the signature; SPTPA1, the 3^*rd*^ gene of the signature, appeared in a less relevant node. The most interesting finding is that TNF-alpha is directly and significantly regulated by IFNK, a gene that has not been previously related with cancer in literature. This suggest that the signature is able to reveal genes that, not being previously related with cancer, may play a significant role in it.

When other genes of the signature not previously related with cancer are inspected along the 19 IPA networks, most of them appeared in peripheral positions, supporting their low beta value, which opens the possibility of studying the putative role of those genes in cancer. Since PCNAP1 is not a gene but a pseudogene, it is not striking that none of the networks contained information about it: usually, pseudogenes are not studied in laboratories. However, since more and more pseudogenes are reported to be involved in cancers in literature [34], we can hypothesize that PCNAP1 is expressed in relation to cancer or cell death, probably due to its genomic context or its behaviour as a lncRNA, as in [34]. Hence, new research should be focused on this pseudogene and cancer to explain why PCNAP1 appeared in a relevant 19^*th*^ position in the gene signature.

In conclusion, the learning approach for the signature is not only revealing genes with important but disregarded roles in cancer, but also genes that should play an unknown or collateral role in cancer.

## Conclusions

In this paper we have proposed the BLASSO predictive model, a new linear *l*_1_-regularization model that incorporates prior biological knowledge into the gene profiles to enrich them with data related to a given target problem. The linear nature of the proposed model makes it highly interpretable as well as it is of benefit to any subsequent biological analysis. Furthermore, *l*_1_-regularization supplies the model with a feature selection mechanism that not only allows the model to avoid over-fitting but, what is even more important, it allows the model to reveal genetic signatures involved in complex human traits.

In order to have a preliminary view of the capabilities of BLASSO in terms of its efficacy as a classifier and also in terms of the stability of the genetic signatures that it supplies, we have first used artificially generated data to validate the model by comparing it with a standard baseline LASSO model with homogeneous priors. Following a 10-fold cross-validation strategy, we have shown how BLASSO outperforms the baseline model in both aspects: it got higher efficacy rates as well as more robust biomarkers than those obtained with LASSO.

Once the model has been validated in an artificial-data scenario, we have used real breast cancer data to test BLASSO, although future work will consider other types of cancer to test the efficacy of BLASSO in different scenarios. For this purpose, we have followed two different approaches that we named *Gene-specific* and *Gene-disease*. For the former, we used the PubTator public repository to supply the gene profiles with information regarding the number of citations in general for each given gene, while for the latter the information obtained from PubTator for each given gene was restricted to the number of citations related specifically to breast cancer. By following these two approaches, we have explored the capabilities of BLASSO in predicting breast cancer outcomes and supplying genetic signatures for this given disease.

We have used 10-fold cross-validation with 100 repetitions for model assessment by tuning the two given hyper-parameters of the BLASSO: the regularization rate (*λ*) and the degree of smoothness of the individual-gene regularization (*ε*). In terms of efficacy rates (AUC) of the classifiers, our results have shown how for both approaches above it is possible to find a parameterization of the models for which the baseline estimation is outperformed. The AUC values obtained by BLASSO supposed a quantitative and qualitative improvement, pushing the AUC up to 0.7 and 0.69 in contrast to 0.65 achieved by LASSO. Moreover, these performance were statistically significant getting very low *p*-values after applying a Wilcoxon signed rank test. In addition, the improvement obtained was achieved using in average less genes than the ones retained by LASSO. Regarding the stability of the genetic signatures found, both proposed approaches outperform the baseline model in terms of the robustness index defined, highlighting how the *Gene-specific* approach was able to find genetic signatures 66% more robust in average (RI of 0.15 compared to 0.09 obtained by LASSO).

Finally, the functional analysis of the genetic signature found by the proposed model (when BLASSO with the *Gene-disease* approach was estimated to the complete BRCA dataset) has revealed some important findings. As expected, the incorporation of prior biological information into the gene expression profiles in the dataset has given rise to a genetic signature that bears significant biological information related to the target problem. In this sense, not only have the breast cancer pathways and networks been pinpointed by the biomarkers, but also have other pathways and networks related to cancer in general been included in the genetic signature. This means that other not-yet or less studied genes related to breast cancer could have been captured as biomarkers. In this vein, the most remarkable cases are the gene IFNK and the pseudogene PCNAP1, that have been both significantly included in the genetic signature found by BLASSO but their implication to breast cancer remains unknown for the moment: the former seems to have a collateral relationship with cancer, while the latter plays an unknown role in this disease.

## Abbreviations

AUC: area under receiver operating characteristic curve
BLASSO: biological least absolute shrinkage and selection operator
BRCA: breast cancer dataset
CI: confidence intervals
FS: feature selection
IFNK: interferon kappa
IPA: ingenuity pathway analysis
LASSO: least absolute shrinkage and selection operator
ML: machine learning
PCNAP1: proliferating cell nuclear antigen pseudogene 1 in human
RI: robustness index
RNA-Seq: ribonucleic acid - sequencing
TCGA: the cancer genome atlas
WebGestalt: web-based gene set analysis toolkit
